# Defining major trauma: a literature review

**DOI:** 10.29045/14784726.2019.06.4.1.22

**Published:** 2019-06-01

**Authors:** Lee Thompson, Michael Hill, Gary Shaw

**Affiliations:** North East Ambulance Service NHS Foundation Trust; Northumbria University; Northern Trauma Network: ORCID iD: https://orcid.org/0000-0002-0820-1662; Northumbria University; North East Ambulance Service NHS Foundation Trust

**Keywords:** injury, major trauma, mechanism, pre-hospital

## Abstract

**Introduction:**

Major trauma in the elderly population has been increasingly reported over the past decade. Compared to younger populations, elderly patients may experience major trauma as a result of low mechanisms of injury (MOIs) and as a result, existing definitions for ‘major trauma’ should be challenged.

This literature review provides an overview of previous conceptualisations of defining ‘major trauma’ and considers their utility in relation to the pre-hospital phase of care.

**Methods:**

A systematic search strategy was performed using CINAHL, Cochrane Library and Web of Science (MEDLINE). Grey literature and key documents from cited references were also examined.

**Results:**

A total of 121 articles were included in the final analysis. Predominantly, retrospective scoring systems, such as the Injury Severity Score (ISS), were used to define major trauma.

Pre-hospital variables considered indicative of major trauma included: fatal outcomes, injury type/pattern, deranged physiology and perceived need for treatment sequelae such as intensive care unit (ICU) admission, surgical intervention or the administration of blood products.

Within the pre-hospital environment, retrospective scoring systems as a means of identifying major trauma are of limited utility and should not detract from the broader clinical picture. Similarly, although MOI is often a useful consideration, it should be used in conjunction with other factors in identifying major trauma patients.

**Conclusions:**

In the pre-hospital environment, retrospective scoring systems are not available and other variables must be considered. Based upon this review, a working definition of major trauma is suggested as: ‘A traumatic event resulting in fatal injury or significant injury with accompanying deranged physiology, regardless of MOI, and/or is predicted to require significant treatment sequelae such as ICU admission, surgical intervention, or the administration of blood products’.

## Introduction

Major trauma is a leading cause of death, with 5.8 million people dying annually worldwide (Bouzat et al., 2015). Kehoe, Smith, Edwards, Yates and Lecky (2015) state that major trauma is perceived to be a younger person’s disease as it is a leading cause of death and disability for those aged less than 40 years. However, it is now recognised that an increasing proportion of major trauma patients are elderly, with significant injuries from relatively low mechanisms of injury (MOIs) such as a fall from standing height.

**Figure 1:**
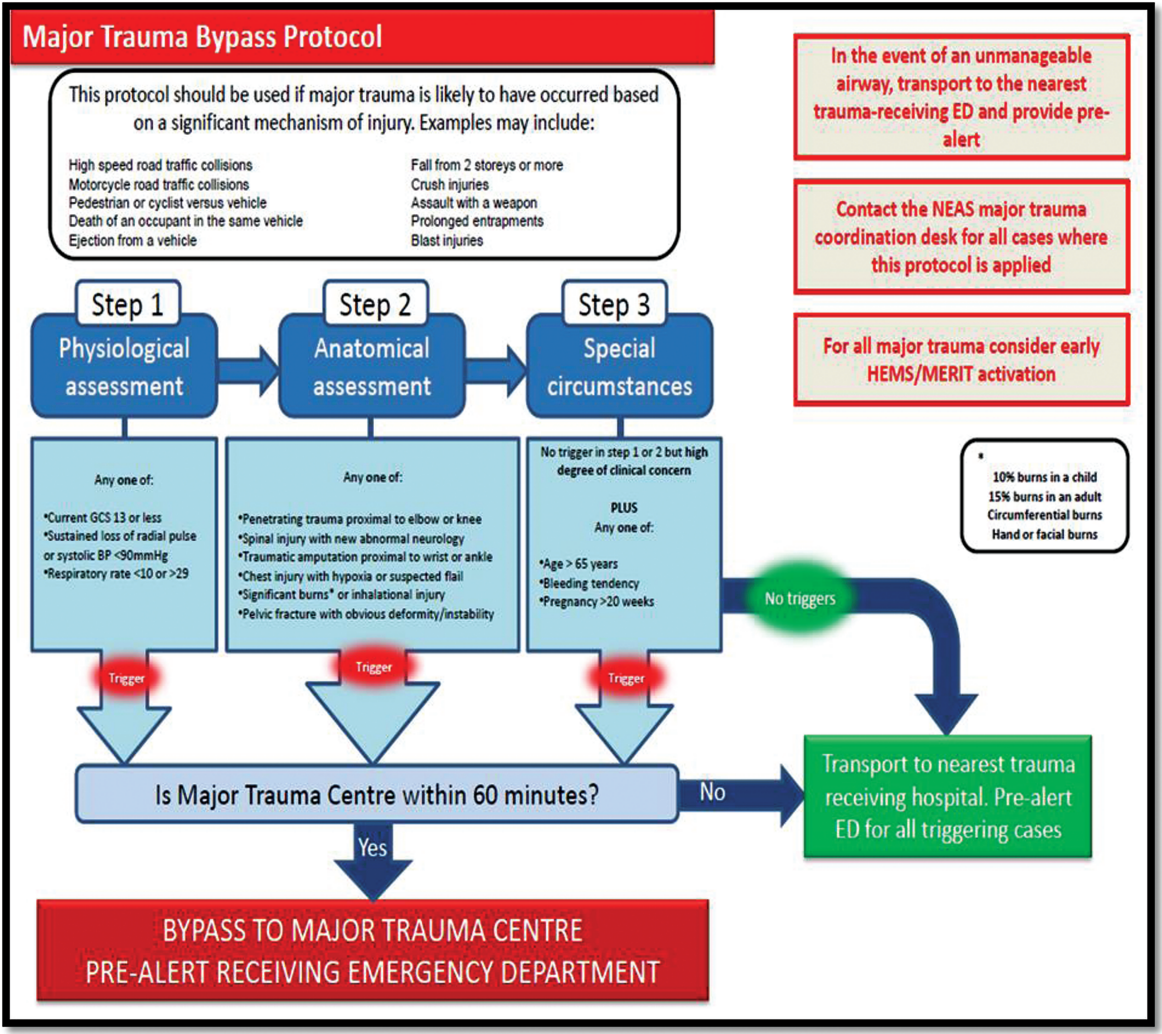
Figure 1. Major trauma bypass protocol.

Within the Northern Trauma Network (NTN) it has been debated as to what defines a patient as having ‘major trauma’. To access specialist care at a major trauma centre (MTC) a bypass tool is utilised. The major trauma bypass protocol used by clinicians within the NTN utilises physiology, anatomical injuries and special circumstances to identify ‘major trauma’. However, the patient must first have a significant MOI which often excludes the older adult falling from less than two metres (see Figure 1). Each individual trauma network in the United Kingdom has a bespoke bypass protocol for their region to account for local idiosyncrasies, but there is little to differentiate each tool.

Data from the Trauma Audit Research Network (TARN), a national trauma registry for England and Wales, highlight that, within the NTN, 56% of TARN eligible patients between April 2012 and March 2017 had significant injuries due to falls less than two metres.

The Injury Severity Score (ISS) is often used by trauma registries such as TARN to describe the aggregation and severity of injury. TARN data must be submitted within 25 days post-incident and scores are then attributed to patients based on that data. Alberdi, Garcia, Atutxa, Zabarte and Trauma and Neurointensive Care Work Group of the SEMICYUC (2014) state that there is no worldwide standardised definition for major trauma and that retrospective scores (such as ISS) are commonly used to quantify the severity of injury.

Retrospective injury scores are not available during the pre-hospital phase of care. In the absence of scoring systems, and acknowledging the significance of considerable injury originating from low energy mechanisms, there are conflicting views of what defines major trauma and this has generated our research question: ‘What is the definition of major trauma?’.

### Aim

This literature review aims to provide an overview of existing definitions of ‘major trauma’ and considers their utility in relation to the pre-hospital phase of care.

## Methods

The PRISMA reporting method was used throughout the review (Moher, Liberati, Tetzlaff, Altman, & The Prisma Group, 2009). The patient group (P) included all trauma patients, the intervention (I) was the explicit definition of major trauma, with a comparison (C) consisting of all definitions of major trauma and finally, the outcome (O) was a consensus of the definition of major trauma. A liberal approach to study designs (S) was adopted, with cohort studies, case reports, systematic reviews and meta-analysis, as well as expert opinion and commentaries, all considered.

A systematic search of the literature was performed using CINAHL, Cochrane Library and Web of Science (MEDLINE). No date ranges were set during the search criteria.

Inclusion criteria were developed with the assistance of a research librarian from Northumbria University, and included all patients, regardless of age, with Boolean search terms ‘major’ and ‘trauma’ with the truncation ‘defin*’ to capture all the variations of the word definition (define, defined, definitions, etc.). The additional criteria of a) text in English and b) peer reviewed were also incorporated. No other inclusion/exclusion criteria were considered.

All abstracts identified within the search were reviewed by a single researcher (LT) to ensure the literature contained discussion around the definition of major trauma. A shortlist of abstracts were reviewed in full text and included. The sole criterion for inclusion in the final list was the presence of a definition of major trauma within the body of the article. The intention therefore was to obtain and review multiple perspectives of major trauma definition.

The search was complimented by additional texts identified via reference lists of the original articles and relevant grey literature known to the authors.

## Results

**Figure 2:**
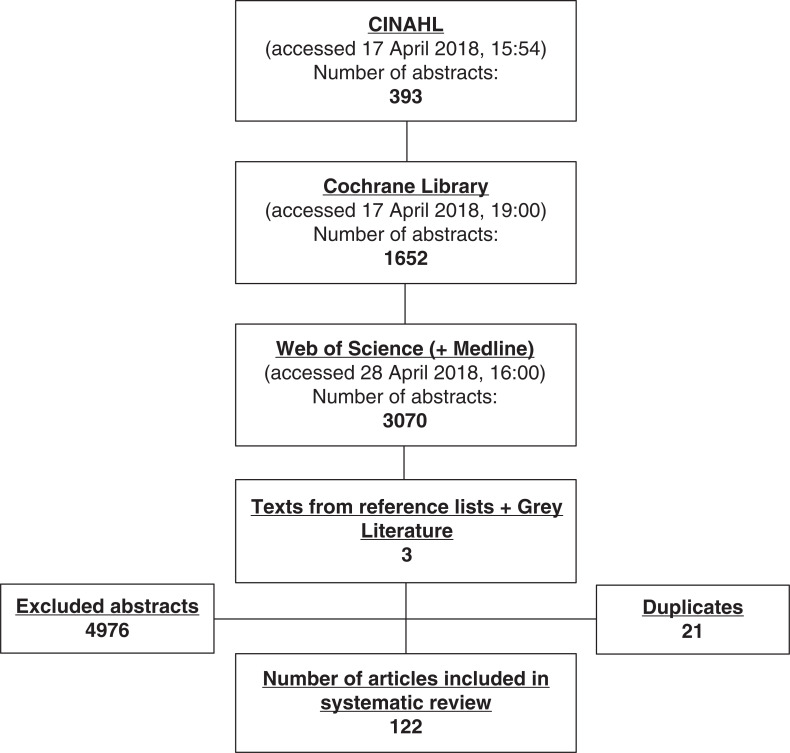
Figure 2. Results of literature search and selection process.

The initial search identified 5118 abstracts which were reviewed by a single researcher (LT) to obtain a short list of relevant documents. Those texts that did not provide a clear definition for major trauma (4976) were excluded from the review, as were duplicates (21). A total of 121 texts were considered to be appropriate for this review and read as full texts (see Figure 2).

Each document was examined to determine variables used in the definition of major trauma. Many documents used multiple factors to define major trauma. NVivo qualitative data analysis software (QSR International Pty Ltd., Version 11, 2015) was used to code the texts and identify trends and commonality of definitions across all the literature reviewed. Table 1 summarises all the identified individual variables that were used within the literature to define major trauma.

**Table 1.** Summary of individual variables identified within the literature for defining major trauma.

Criteria for defining major trauma	*(n)*
Retrospective injury scores (inc. ISS, NIS, AIS, etc.)	109
Injury Severity Score (ISS)	103
Fatal outcome	21
Injury type/pattern	16
Intensive Care Unit (ICU) admission	12
Requires surgical intervention	12
Haematocrit decrease	7
Abbreviated Injury Scale (AIS)	6
New Injury Severity Score (NISS)	6
Requiring ventilation	5
Mechanism of injury (MOI)	4
Receiving blood products	4
Deranged physiology	4
Trauma Injury Severity Score (TRISS)	1
Revised Trauma Score (RTS)	1
International Classification of Diseases-derived ISS (ICISS)	1
Hospital Trauma Index ISS (HTI-ISS)	1
Paediatric Trauma Score (PTS)	1
Pre-hospital index greater than 3	1

Some texts used combinations of criteria but most often, they used retrospective scoring systems such as ISS, with the common consensus that a score of greater than 15 was the standard for defining major trauma.

Injury type or pattern was a key definition for many articles that also provide data for retrospective scoring methods, but they need to be appropriate for pre-hospital clinicians (Table 2). Articles by Blacker and Wijdicks (2004), Furrer et al. (1995), Paffrath, Lefering, Flohe and TraumaRegister DGU (2014), Rowell et al. (2011), Sapan et al. (2016), Shahim, Cameron and McNeil (2006) and Stuke et al. (2013) all use multiple injuries/fractures as part of their definition of major trauma. Several also state the need to have deranged physiology in addition to multiple injuries to qualify as major trauma (Barrera et al., 2013). Other articles are more specific in mentioning individual injuries such as pelvic fracture, spinal fractures and chest or abdominal injuries (Bressan et al., 2015; Burbridge, Groot, Oleniuk, Taranger, & Barrett, 1991; Cox et al., 2011; Shahim et al., 2006; Stuke et al., 2013; Voth, Lustenberger, Auner, Frank, & Marzi, 2017). It is important to note that these texts almost exclusively report case series that examine specific sub-groups of patients who experience polytrauma. Although they list individual or multiple injuries in their definition of major trauma, they also state other variables such as ISS.

**Table 2.** Potential pre-hospital variables identified as defining major trauma.

Variables	*(n)*
Fatal outcome	21
Injury type/pattern	16
Intensive Care Unit (ICU) admission	12
Requires surgical intervention	12
Mechanism of injury (MOI)	9
Haematocrit decrease	7
Requiring ventilation	5
Pre-hospital index	4
Receiving blood products	4
Deranged physiology	2
Revised Trauma Score (RTS)	2
Paediatric Trauma Score (PTS)	1

Haematocrit point of care testing was identified in seven articles, but is not routinely available in the NTN region for pre-hospital crews, although our Helicopter Emergency Medical Services (HEMS) carry blood products. The need for blood products can be anticipated by pre-hospital teams and, to a limited extent, so can the need for surgical intervention and intensive care unit (ICU) admission, although this may be difficult in some instances. Within the NTN region, the criteria for requiring blood products is the same for tranexamic acid (TXA) administration and therefore pre-hospital teams could use the administration of TXA as clear indication for needing blood products.

The Revised Trauma Score (RTS) (Cummings & Mayes, 2007) and pre-hospital index (Lavoie, Emond, Moore, Camden, & Liberman, 2010) use deranged physiology as indicators for major trauma and are already part of our existing regional major trauma bypass protocol and therefore appropriate to pre-hospital teams. The Paediatric Trauma Score (PTS) (Narotam, Budonrappa, Raynor, Rao, & Taylon, 2006) also uses deranged physiology (systolic blood pressure and mental status) in addition to weight, airway status, fractures and wounds, as part of the scoring matrix. Although specific to paediatrics, these factors can be applied to defining major trauma in the pre-hospital environment.

There were nine articles from the 121 texts identified in the review that highlight MOI when attempting to define major trauma (see Table 3).

**Table 3.** Defining major trauma using mechanism of injury (MOI).

Authors	Aim	Sample	Method	Location	Definition of ‘major trauma’/comments	OCEBM Level of Evidence score*
Barrera et al. (2013)	To assess the effects of thromboprophylaxis in trauma patients on mortality and incidence of deep vein thrombosis and pulmonary embolism. To compare the effects of different thromboprophylaxis interventions and their effects according to the type of trauma.	Sixteen RCT studies were included in this systematic review (n = 3005).	Systematic review of randomised controlled trials.	Various, including UK, Continental Europe and USA.	For purposes of inclusion major trauma defined broadly, e.g. physiological: penetrating or blunt trauma with more than two organs and unstable vital signs; anatomical: people with an ISS higher than 9; mechanism: people who are involved in a ‘high energy’ event with a risk for severe injury despite stable or normal vital signs. *Comments:* Authors did not seek to define major trauma as a primary objective of this study, but only as an operational means of assessing the clinical efficacy of thromboprophylaxis in trauma patients. The adoption of a wide operational definition is perhaps a function of the need to include sufficient articles in this systematic review.	1a
Bond, Kortbeek and Preshaw (1997)	To explore if combined trauma scores improve the sensitivity and specificity over that of individual scores.	3147 trauma incidents which identified the nature of injury (MOI).	Prospective cohort study combining PHI and ISS where MOI is identified.	City of Calgary, Canada.	ISS > 16. Authors concluded that combining pre-hospital index with mechanism can identify ISS > 16. Authors note that not all ‘major trauma’ can be identified this way.	2b
Boyle, Smith and Archer (2008)	To determine if MOI alone is a useful predictor of major trauma in pre-hospital trauma triage.	4571 trauma incidents in which MOI was used as a basis of assessment.	Retrospective cohort study using secondary data analysis of existing trauma case records.	Victoria, Australia.	Authors claim that individual MOI criteria have no clinical or operational significance in pre-hospital trauma triage of patients who have an absence of physiological distress and significant patterns of injury.	2b
Cooper, Yarbrough, Zonesmith, Byrne and Norcross (1995)	To investigate the appropriateness of MOI as an exclusive indicator for trauma centre triage.	112 clinician questionnaires.	A prospective cohort study utilising clinician reported data questionnaires.	South Carolina, USA.	MOI alone had a positive predictive value of only 6.9%. MOI may not, by itself, justify bypass of local hospitals in favour of trauma centres.	2b
Kubat, Buiskool and van Suylen (2015)	To define minor- and major-trauma and to analyse the likelihood of fatal outcome if VAI is present.	150 publications reviewed resulting in (n = 241) trauma cases where VAI was present.	Narrative review. Secondary data analysis of retrospective case reports.	Various, including USA, Canada, Japan, UK, Continental Europe.	The authors employ no concrete definition of major trauma but advise that MOI (with radiological screening) allows for differentiation between major and minor trauma.	4
Lossius, Rehn, Tjosevik and Eken (2012)	To investigate the effects of different definitions of major trauma on perceived over- and under-triage rates.	‘Approximately’ 360 cases.	Retrospective cohort study using secondary data analysis of existing trauma case records.	Stavanger, Norway.	Authors identify ISS > 15 and NISS > 15 as defining major trauma but also identify extended definitions, which include the mechanism of proximal penetrating injury. Authors claim that defining major trauma in terms of mechanism alone drastically increases risk of over-triage.	2c
Magnone, Ghirardi, Ceresoli and Ansaloni (2017)	To analyse the association between MOI and ‘major trauma’ as defined by ISS > 15.	1575 case records.	Retrospective cohort study using secondary data analysis of existing trauma case records.	Bergamo, Italy.	Over half of patients taken to trauma centres based on mechanism alone are discharged from the emergency department. Authors acknowledge the need for separate protocols for older adults.	2b
Potter, Kehoe and Smith (2013)	To assess the sensitivity of the Wessex Triage Tool against cases where ISS > 15.	171 TARN database records.	Retrospective cohort study using secondary data analysis of existing TARN database records.	Plymouth, UK.	Authors identify ISS > 15 as definitive of major trauma but state that pre-hospital triage tools using MOI would exclude many defined major trauma patients, especially older adults.	2b
Stuke et al. (2013)	To review a single centre experience with past and present national triage criteria to determine which MOI predicts trauma centre need.	3569 case records.	Retrospective cohort study using secondary data analysis of existing trauma case records.		Significant predictors of Trauma Centre Need included death in the same passenger compartment, ejection from vehicle, extrication time of > 20 minutes, fall from > 20 feet, and pedestrian thrown/runover.	2b

*Oxford Centre for Evidence-based Medicine (2009).

Barrera et al. (2013) are the only authors to use a ‘high energy event’ with a risk of severe injury as a definition for major trauma. No other articles use MOI to define major trauma. However, Bond, Kortbeek and Preshaw (1997) use mechanism in combination with pre-hospital index to identify major trauma with an ISS > 16 but recognise this may exclude some major trauma.

Lossius, Rehn, Tjosevik and Eken (2012) specifically use the mechanism of proximal penetrating trauma in their extended definition of major trauma. Stuke et al. (2013) do not use mechanism to define major trauma but use death in the same passenger compartment, ejection from vehicle, extrication time of more than 20 minutes, fall from more than 20 feet and pedestrian thrown/runover as indicative of trauma centre need.

The majority of articles that discuss MOI in defining major trauma either suggest that mechanism does not identify all major trauma (Bond et al., 1997; Cooper, Yarbrough, Zonesmith, Byrne, & Norcross, 1995; Potter, Kehoe, & Smith, 2013; Stuke et al., 2013) or that it over-triages major trauma (Lossius et al., 2001; Magnone, Ghirardi, Ceresoli, & Ansaloni, 2017). For example, Boyle (2007) categorically states that MOI should not be used to identify major trauma and that, in the absence of deranged physiology or injury pattern, it has no clinical significance. Magnone et al.’s (2017) MOI article is interesting in that they state that older adults should have their own field triage protocol to identify those requiring expertise to manage their outcomes.

## Discussion

The main method for defining major trauma is by using injury severity scoring systems. Although these are retrospective scores, they are essentially an aggregation of the patients’ injuries.

The ISS (Baker, O’Neill, Haddon, & Long, 1974) originated in order to identify (and provide some equivalence between) anatomically different injuries of equal severity. The score was developed on the basis of road traffic collision (RTC) data for 2128 motorists, passengers, pedestrians and other road users over two years. Data were first classified in accordance with an existing Abbreviated Injury Scale (AIS) score for each body region and severity of injury (see Table 4) ranging from 1 (minor) to 6 (non-survivable). Notably, ISS is calculated retrospectively after patients have undergone imaging and interventions to identify and potentially manage injuries. MTCs have 25 days and trauma units (TUs) have 40 days to upload patient data to TARN, which then calculates the scores. The pre-dominant score from each body region (head or neck, face, chest, abdomen, extremity or pelvis, external) is squared and the three highest scoring body regions added together to calculate the ISS (Trauma Audit Research Network, 2018). This is a complex process and not all injuries will be apparent or easily identified in the pre-hospital phase of care.

**Table 4.** Abbreviated Injury Scale.

1	Minor
2	Moderate
3	Serious
4	Severe
5	Critical
6	Non-survivable

However, retrospective scores are not helpful for a pre-hospital clinician trying to triage the patient they are caring for. Furthermore, even at its inception, it was noted that discrepancies in the use of ISS existed between receiving hospitals and the ISS was noted to prove particularly problematic when considering children and older people. In relation to the latter point, the original authors acknowledged that ‘increased mortality in the elderly is most pronounced when the injuries are *least* severe’ (Baker et al., 1974, emphasis in original).

An ISS ≥ 15 is the predominant definition for major trauma from the literature highlighted within this review. It is a universally accepted measure of major trauma based on injury types, body area and aggregation of injuries. This scoring system was not designed to prospectively identify possible injuries, and therefore MOI, physiology, haematocrit levels and age become irrelevant when providing a score for identified injuries. With this knowledge, it could be argued that all other variables be excluded from defining major trauma. As already stated, these scoring systems are complex and not easily applied in the pre-hospital phase with limited diagnostic equipment, and other criteria may be appropriate to define major trauma for this phase of care. While progress in medical diagnostic technologies and classification systems such as ISS have (arguably) improved healthcare delivery (OECD, 2018), authors such as Lu (2016) have argued that this statement cannot be observed to be universally true. As clinicians become increasingly reliant upon scoring and measurement to inform (and perhaps even shape) clinical decision-making, it is arguable that there is a subsequently decreasing reliance upon clinical examination and history taking – potentially deskilling practitioners (Verghese & Horwitz, 2009).

Epner, Gans and Graber (2013) have suggested that an orthodox account of the role of diagnostic scoring in clinical situations would suggest that: a) diagnosis arises primarily from history and physical examination; and b) diagnostic testing/scoring is used merely to confirm clinical diagnosis. However, diagnostic testing and measurement have expanded in parallel with technological advances, and arguably this has been accompanied by a ‘rising tide’ of diagnostic testing in areas such as pre-hospital care. In most instances, the value of such testing is assessed in its own terms, for example measures of laboratory efficiency and internal consistency, rather than in terms of actual patient outcomes.

Multiple injuries are a common feature of defining major trauma but it is noted that individual injuries in the presence of deranged physiology can also be indicative of major trauma. Fatalities due to trauma are also regularly referred to in the literature as major trauma.

A fatal outcome from trauma can be applied to any setting and should therefore be applied to the pre-hospital definition of major trauma. However, it should also be noted that fatalities of medical origin are often the primary causal factor of, for example, RTCs. The practice of recording these fatalities in trauma statistics probably represents a significant source of data contamination, as any concurrent injuries – however severe – are often not the cause of death.

The difficulties in dealing with the cumulative effects of poly-trauma – where ISS was recorded in accordance with the most severe injury observed, regardless of the extent of more minor concurrent injuries – were only partially resolved by use of the scoring system outlined above. Further problems included the fact that no sensitivity or specificity analysis was undertaken in relation to this scale. Moreover, the scale was based exclusively upon RTC data, and therefore trauma as a consequence of high energy transfer. While these data may therefore usefully translate into similar MOIs, such as falls from a significant height, their use in mechanisms such as stabbing, gunshot wounds or low energy falls may be more problematic.

Prediction of potential ICU admission, surgical intervention, need for blood products or ventilation (predicted or ongoing) are all potentially identifiable by pre-hospital teams and routinely used to define major trauma in the literature.

The literature indicates that deranged physiology (low blood pressure, reduced consciousness, low or high respiration rates) is indicative of the body’s response to major trauma and is commonly identified within an initial assessment (Kim et al., 2017). Deranged physiology is also a key component of the pre-hospital index, RTS and PTS.

Within the NTN, a significant MOI must be considered when using the major trauma bypass protocol (Figure 1) but is not a trigger to attend an MTC in its own right. This has led to some debate as to what to consider major trauma, if the trauma arises from minor mechanisms such as a fall from standing. Surprisingly, MOI is explicitly referenced in the literature when defining major trauma only to highlight that, in isolation, mechanism is not appropriate to identify or define major trauma (note how this is embedded into ISS through its historic development).

### Limitations

Within this literature review all articles were identified and assessed using an eligibility criterion with obvious heterogeneity in patient groups and variables. To allow for the reproduction of this systematic narrative review, the method has been carefully described. Only peer-reviewed articles have been used.

## Conclusions

The most common definition of major trauma in contemporary and historical use is that of ‘an ISS greater than 15’. However, in the pre-hospital environment, retrospective scoring systems are not available and other variables must be considered. Based upon this review, a working definition of major trauma is suggested as: ‘A traumatic event resulting in fatal injury or significant injury with accompanying deranged physiology, regardless of MOI, and/or is predicted to require significant treatment sequelae such as ICU admission, surgical intervention or the administration of blood products’.

## Acknowledgements

The researchers acknowledge the contributions of Professor Fiona Lecky for her mentorship throughout this project, and Wilma Harvey-Reid for her valuable time in proofreading and manuscript layout.

## Author contributions

All authors have read and approved the final manuscript.

## Conflict of interest

None declared.

## Ethics

Not required.

## Funding

Small grant winner, sponsored by the College of Paramedics.
